# Antioxidant Response as a Candidate Prognostic Factor for Dengue Hypotensive and Hemorrhagic Complications: Results from a Nested Case-Control Study in Colombia

**DOI:** 10.3390/tropicalmed10010014

**Published:** 2025-01-04

**Authors:** Anyela Lozano-Parra, Víctor Herrera, Luis Ángel Villar

**Affiliations:** 1 Grupo de Epidemiología Clínica, Escuela de Medicina, Universidad Industrial de Santander UIS, Calle 9 Carrera 27, Bucaramanga 680002, Colombia; anyela.lozano@correo.uis.edu.co; 2 Centro de Atención y Diagnóstico de Enfermedades Infecciosas (CDI), Fundación INFOVIDA, Cra. 37 No. 51-126, Bucaramanga 680003, Colombia; direccioninvestigacion@cdi.net.co

**Keywords:** severe dengue, oxidative stress, glutathione peroxidase, superoxide dismutase, total antioxidant status

## Abstract

Dengue infection has been associated with oxidative stress (OS) induction; however, whether such a response predicts the development of complications remains unknown. We conducted a case-control study (1:2 ratio) nested within a cohort of febrile patients with a presumptive or confirmed diagnosis of dengue. Incident cases were patients who developed hypotension or severe bleeding during the follow-up, whereas controls did not. Total antioxidant status (TAS), superoxide dismutase (SOD), and glutathione peroxidase activity (GPx) were quantified in serums obtained ≤96 h from disease onset. The association between each biomarker and complications was evaluated by estimating adjusted odds ratios (ORs) using logistic regression. We evaluated 132 patients (median age: 19.0 years; 58.2% males). TAS and SOD were higher among cases than controls (2.1 versus 1.7 mM and 6.7 versus 6.0 U/mL, respectively), and the opposite was observed for GPx (128.1 versus 133.7 mmol/min/mL); however, none of these contrasts reached statistical significance. In the multivariate analysis, higher levels of TAS and SOD were associated with a higher likelihood of complications up to 3.5 mM (OR = 2.46; 95%CI: 1.10–5.53) and 8.0 U/mL (OR = 1.69; 95%CI: 1.01–2.83), respectively. GPx did not show an association with hypotension or severe bleeding. Our results suggest that the induction of OS during the acute phase of dengue infection might be a prognostic factor of hypotensive and hemorrhagic complications.

## 1. Introduction

The disease caused by dengue virus (DENV) is endemic in more than 100 countries in tropical and subtropical regions [1]. There has been a drastic increase in the incidence of dengue fever in the Americas from 2003 to 2013 [2]. The introduction of the Zika virus in 2016 elicited a meaningful reduction in DENV transmission [3]. Nevertheless, in 2019, DENV reappeared, and its transmission was similar to the outbreak in 2013 [4]. Dengue disease tends to be a self-limiting febrile illness. However, hospitalized patients have a 5–10% risk of progression to severe dengue, and up to 5% of cases may be fatal [1,5]. The severe form of the disease is characterized by plasma leakage, hemorrhaging, and shock [6]. Risk factors of severe DENV include serotype, secondary infections, and some individual conditions, like comorbidities and genetic factors [6,7].

Currently, it is unclear which patients may develop complications. Therefore, it is crucial to explore early prognostic biomarkers of severe dengue. Such biomarkers, if determined during the first hours of the disease, could aid clinicians in stratifying the risk of progression toward severity and implementing more efficient, preventative interventions accordingly. Oxidative stress has been associated with the pathogenesis of various diseases, including viral infections [8]. Reactive oxygen species (ROS) generation could stimulate the secretion of proinflammatory mediators and trigger apoptosis in endothelial cells [9,10]; however, this and other effects are mainly counter-regulated by antioxidants such as catalase, glutathione, glutathione peroxidase (GPx), and superoxide dismutase (SOD) [11]. The imbalance between ROS and the antioxidant response can potentially increase vascular permeability, a hallmark of severe dengue [10,12].

Observational studies conducted in patients with dengue have reported alterations in oxidative activity [13,14,15,16,17]. These include changes in the activity of glutathione peroxidase (GPX), the concentration of superoxide dismutase (SOD), and total antioxidant status (TAS) during the acute phase of de the disease. However, there is limited and inconclusive evidence linking biomarkers of oxidative stress and dengue complications in part due to the heterogeneity in the definition of outcomes, the duration of disease at which they have been determined, and the nature of comparison groups across studies. Our study seeks to address some of these gaps by evaluating the prognostic potential of TAS, SOD, and GPx for predicting hypotensive and hemorrhagic complications in dengue patients using a nested case-control study.

## 2. Materials and Methods

### 2.1. Study Design

We conducted a nested case-control study within a cohort of febrile patients with no apparent infectious focus after undergoing a physical examination. Participants were enrolled in ambulatory health care services in five cities with high DENV transmission in Colombia (Bucaramanga, Piedecuesta, Floridablanca, Palmira, and Barranquilla) from May 2009 to April 2011. Those municipalities were selected to represent the spectrum of urbanization across diverse geographical endemic regions of the country whose high average incidence rates during the preceding decade (147.3–559.9 per 100.000 inhabitants) would maximize the likelihood of identifying severe cases of DENV infection [18]. Eligible participants of the cohort were ≥5 years of age and were diagnosed with dengue acute disease of <96 h of symptoms’ onset before enrolment. We excluded patients with a history of cancer, acquired immunodeficiency syndrome, chronic kidney disease, cardiac disease, or hematologic disorders; those who used corticosteroids in the last 8 days, had major bleeding (hematemesis, hematuria, hematochezia or melena) or clinical signs of plasma leakage (pleural effusion, ascites, and hypotension) at enrollment; and those recruited in inpatient services.

Trained physicians obtained demographic and clinical data at enrolment. Acute and convalescent (7–15 days) blood samples were collected to determine a complete blood count (enrolment) and to perform diagnostic tests. Participants were followed daily until the seventh day of the disease to collect clinical information (symptoms and signs) and to determine the microhematocrit. We defined a confirmed dengue case according to a diagnostic algorithm, which included IgM/IgG seroconversion (shift from negative to positive or a titer increase ≥4) in paired sera, a positive NS1 protein result or viral genome amplification per RT-PCR in acute serum (<96 h post onset of fever) with the use of methods previously described [19]. Patients with a positive result of IgM in any sample (acute or convalescent) were considered presumptive cases of dengue, which, for the purpose of the analysis, were combined with confirmed cases considering the high positive predictive value (96.2%) of a single positive IgM test among symptomatic patients [20]. Additionally, we considered primary DENV infections to be those with a negative IgG result in the acute sample. IgM and IgG antibodies were determined using an enzyme-linked immunosorbent assay (ELISA), specifically Dengue IgM/G Capture ELISA PanBio^®^ (Abbott, East Brisbane, QLD, Australia). The NS1 antigen was quantified with the ELISA NS1 Dengue PanBio^®^ (Abbott, East Brisbane, QLD, Australia). To detect the viral genome, we extracted the RNA with the QIAamp Viral RNA kit (Qiagen, Hilden, Germany) and then performed a reverse transcription polymerase chain reaction (RT-PCR) based on a previously established method [21].

### 2.2. Case-Control Definition

We adopted the dengue case classification suggested by WHO in 2009 [22]: incident cases were patients with dengue infection (presumptive or confirmed) who developed hypotension or severe bleeding during follow-up. Hypotension was defined as the finding of tachycardia and pulse pressure (PP) lower than 20 mmHg, low mean arterial pressure (MAP) for the age, or low systolic blood pressure (SBP) for the age [23]. We considered hematemesis, hematuria, hematochezia, and melena cases of severe bleeding. Controls were randomly selected among patients with presumptive or confirmed dengue infection who did not develop hypotension or severe bleeding during the follow-up [22]. This sampling strategy aimed to minimize selection bias as controls were sampled from the same base population from which cases emerged.

### 2.3. Biomarkers Assays

We performed biomarker assays in acute serum samples (stored at −80 °C) using Cayman^®^ assay kits (Cayman, Ann Arbor, MI, USA) in the Multiskan microplate spectrophotometer (Thermo Scientific, Waltham, MA, USA). We measured glutathione peroxidase activity (GPx. Catalog no. 703102), superoxide dismutase concentration (SOD. Catalog no. 709002), and total antioxidant status (TAS, Catalog no. 709001) as antioxidant biomarkers; however, due to aliquot depletion we could only perform the determination of TAS and SOD in a subsample.

#### 2.3.1. Glutathione Peroxidase

GPx assay is based on a previously established method [24], that measures the activity indirectly by a coupled reaction with glutathione reductase. The oxidation of NADPH to NADP+ is accompanied by a decrease in absorbance at 340 nm. The rate of decline in the absorbance is directly proportional to the GPx in the sample. One unit of GPx is defined as the amount of enzyme that causes the oxidation of 1 nmol of NADPH to NADP+ per minute at 25 °C.

#### 2.3.2. Superoxide Dismutase

We performed the SOD measurement according to the protocol previously established [25]. The assay utilizes a tetrazolium salt to detect superoxide radicals generated by xanthine oxidase and hypoxanthine. One unit of SOD is defined as the amount of enzyme needed to produce 50% dismutation of superoxide radical. We measured the absorbance at 450 nm and expressed the concentration in U/mL.

#### 2.3.3. Total Antioxidant Status

We quantified TAS concentration following the ABTS method [26]. The assay relies on antioxidants’ ability to inhibit metmyoglobin’s oxidation of ABTS to ABTS+. We measured the amount of ABTS+ at 750 nm and expressed its concentration in mM.

### 2.4. Ethical Considerations

The Ethical Review Committee of the Universidad Industrial de Santander approved the study protocol (Acta No. 09, 25 April 2014). We obtained written informed consent from each adult participant, and in the case of children, we obtained their assent and informed consent from their parents or legal guardians.

### 2.5. Data Analysis

We calculated a total sample size of 132 patients (1:2 case-control ratio) to detect a difference equal to or greater than 20% in the concentration of GPx between groups based on absolute concentrations previously reported in patients with dengue [13] with a statistical power of 80% and a type I error of 5%; however, due to aliquot depletion, we could only analyze a subsample of 59 patients for TAS and SOD (complete-case analysis). We described continuous variables by estimating the mean and standard deviation (SD) or the median and interquartile range [IQR] for those not normally distributed, according to the Shapiro-Wilk test. We calculated their absolute and relative frequencies (percentages) for discrete variables. We contrasted means and medians between groups using a student’s t-test and the Kruskal-Wallis test, respectively, and differences in proportions using the chi-square test and, alternatively, the Fisher’s exact test whenever the expected counts in contingency tables were less than five. We explored the shape of the functional relationship between each biomarker and the case-control status using locally weighted regression to identify inflection points. Then, we conducted multiple logistic regression with and without piecewise (using the cut-points previously determined) and estimated odds ratios (ORs) with 95% confidence intervals (95%CI), forcing the adjustment for age but conditioning it by sex, disease duration at enrollment, or infection type (primary versus secondary) only if these covariates were associated to the case-control status at a significance level of ≤10% in the bivariate analysis. There was no further adjustment for warning signs as they could mediate the associations under study. We evaluated the goodness of fit of the models using the Hosmer-Lemeshow test (HL) and selected among them using the Akaike Information Criterion (AIC). Data analysis was conducted using the statistical software Stata/IC, version 14.2.

## 3. Results

The median age of the 132 subjects was 19.0 years (IQR: 13.0, 35.0 years) of whom 67 (58.2%) were males. Cases were younger than controls (medians: 15.0 versus 24.5 years, *p* = 0.007); however, both groups were similar regarding sex distribution, disease duration, and type of infection (Table 1). In terms of warning signs, cases were more likely to report persisting vomiting and mucosal bleeding than controls at baseline: 6.8% versus 0.0% (*p* = 0.035) and 20.5% versus 0.0% (*p* < 0.001), respectively. TAS and SOD were higher among cases than controls, and the opposite was observed for GPx; however, none of these contrasts reached statistical significance (Table 2).

**Figure 1:**
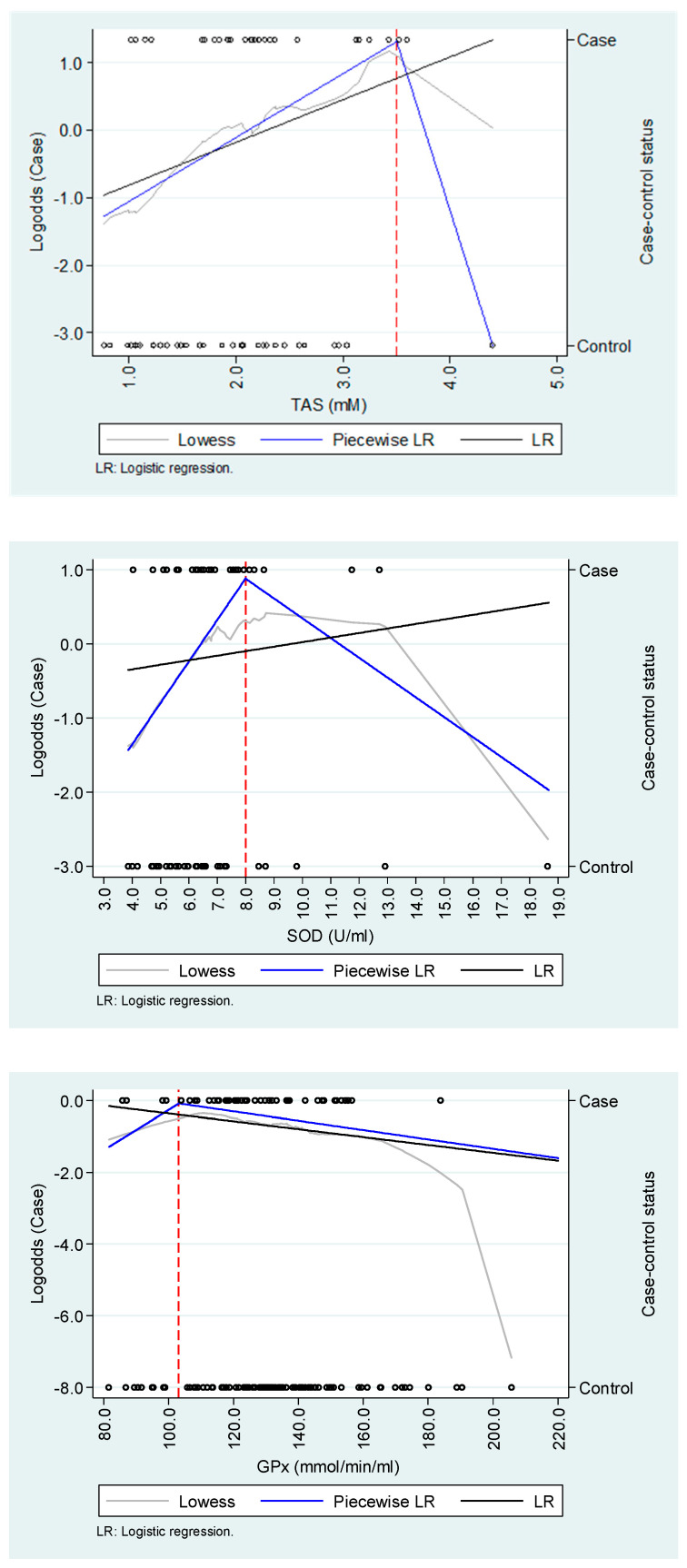
Exploration of the functional relationship between oxidative stress biomarkers and dengue complications. Open circles represent individual observations (cases or controls).

We found evidence of non-linear relationships between TAS and SOD and dengue hypotension or severe bleeding in the explorative analysis; however, they seemed to be highly sensitive to a biologically plausible outlier observation among controls (Figure 1). For TAS and SOD, logistic regression models using a piecewise approach were more parsimonious than those that did not implement it, but the opposite occurred for GPx (Table 3). TAS and SOD were associated with the likelihood of hypotension or severe bleeding up to 3.5 mM (OR = 2.46; 95%CI: 1.10–5.53) and 8.0 U/mL (OR = 1.69; 95%CI: 1.01–2.83), respectively; however, we did not find evidence of a relationship for any of those biomarkers above those cut-points. In any regression model, GPx did not show an association with hypotension or severe bleeding. The results from a sensitivity analysis excluding patients with presumptive DENV infection (6.7% and 9.8% for TAS/SOD and GPx analyses) did not differ qualitatively (i.e., in the functional relationship between exposures and outcomes); however, the association between SOD and complications along the first segment of concentration (<8.0 U/mL) was attenuated and turned non-statistically significant (Supplementary Table S1).

## 4. Discussion

In this nested case-control study, we observed evidence that oxidative stress induction during the acute phase of dengue infection was associated with the incidence of hypotensive and hemorrhagic complications. Specifically, higher levels of the total antioxidant status (TAS) and superoxide dismutase (SOD) but not of glutathione peroxidase (GPx) were associated with a higher likelihood of dengue complications; however, those relationships were exclusively observed for concentrations below 3.5 mM and 8.0 U/mL, respectively.

The literature suggests that oxidative stress plays an important role in the pathogenesis of dengue fever [13,14,15,16,17,26,27,28,29]. During the acute phase of the infection, reactive oxygen species (ROS) are produced due to the release of proinflammatory cytokines [30]. In this scenario, an imbalance between ROS generation and the antioxidant defense system leads to oxidative stress and cellular damage [11,13]. The evidence consistently shows a down-regulation of genes that encode endogenous enzymatic antioxidants and a lower expression of their products in patients with dengue infection as compared to healthy controls; however, the results from the studies are conflicting when contrasting patients across the spectrum of disease severity [27,28]. Further, most of the studies have a case-control design and quantified biomarkers concurrently with the identification of complications, which precludes them from being considered prognostic factors.

Our results show that measured early in the course of the disease, higher levels of the TAS increased the likelihood of progression toward hypotensive and hemorrhagic manifestations. This finding is in alignment with previous observations of higher concentrations of malondialdehyde (MDA), a biomarker of the oxidative stress response, in complicated than uncomplicated dengue patients [17,29,30]. However, our findings seem to conflict with the report of no difference in TAS between those groups early in the course of the disease but lower TAS in complicated patients evaluated at longer disease duration [29]. An explanation for this discrepancy might be that prospectively evaluated, as in our study, patients whose disease onset is characterized by a more intense induction of ROS express a higher TAS as a counterregulatory mechanism, a response that declines during follow-up, leading to complications.

We also quantified the concentration of SOD and GPx, two endogenous enzymatic antioxidants that contribute to the TAS. While the concentration of SOD showed a similar predictive value for dengue hypotensive and hemorrhagic complications as the TAS, GPx was not associated with the likelihood of progression. The first finding is novel and partially supported by the observation of a slightly higher *SOD gene* expression reported by Cherupanakkal et al. in severe as compared to non-severe cases of dengue [27]. Three more studies have evaluated the concentration of SOD in complicated and uncomplicated dengue patients without finding evidence of statistically significant differences between groups [13,15,17]; however, in at least one, the absolute difference was of a similar magnitude as that we observed in our study [13]. Regarding GPx, our results are consistent with those previously reported by Rojas et al. from a cohort study conducted in Colombia [16]. To our knowledge, this is the only longitudinal study that has approached this issue. In addition to the strength derived from its prospective nature, this study showed that a higher baseline concentration of GPx increased the likelihood of spontaneous bleeding at follow-up; however, such a relationship became non statistically significant after adjustment for relevant covariates.

Taken together, our findings are biologically plausive, considering that the dengue virus induces the release of inflammatory cytokines such as IL-1 and TNF-α in infected cells (monocytes, dendritic cells, macrophages, and lymphocytes CD4+ and CD8+), which play a role as pro-oxidants during the acute phase of the disease [31,32]. Furthermore, a positive correlation between TNF-α and MDA, a product of polyunsaturated lipid degradation by ROS, has been reported in complicated but not uncomplicated cases of dengue [30]. The ROS generated during the infection triggers the release of antioxidants molecules, including SOD, GPx, catalase, albumin, ceruloplasmin, ferritin, ascorbic acid, α-tocopherol, β-carotene, reduced glutathione, uric acid, and bilirubin, all of which are included in the measurement of TAS [33]. Considering our results, we propose that the antioxidant response may become overwhelmed and insufficient to control the release of ROS induced by the strong proinflammatory cytokine cascade during the acute phase of dengue in cases that will progress to the severe form of the disease. Moreover, TAS provides a general overview of the antioxidant barrier in plasma, making both TAS and SOD potential prognostic biomarkers of dengue hypotensive and hemorrhagic complications.

Our study has some strengths worth mentioning. First, it is a case-control study nested in a cohort of patients with a diagnosis of dengue, which implies, on the one hand, that cases of hypotensive and hemorrhagic complications were prospectively ascertained (i.e., incident cases) and, on the other, that controls were chosen from the same population that cases which minimizes the risk of selection bias. Secondly, the adjudication of outcomes was performed by trained clinicians blinded to the concentration of the biomarkers of antioxidant status, minimizing the risk of information bias. Thirdly, we tested for and adjusted the associations under study by biologically and clinically relevant confounders. This study also has limitations. Firstly, we confirmed DENV infection through a positive IgM result from a single sample in 4 (6.7%) of patients in the TAS/SOD analysis and 13 (9.8%) of patients in the GPx analysis. Given the high positive predictive value of IgM in relation to virus isolation and/or a fourfold increase in IgM titers from convalescent to acute samples (96.4%) [20], the expected classification error in defining our study sample was minimal (less than one patient in the sample). Secondly, we exclusively measured biomarkers of the antioxidant response at the time of admission of participants to the study, which limits our understanding of the dynamic interplay between pro-oxidants and antioxidants as the disease progresses. Thirdly, although cases and controls were similar regarding the prevalence of primary versus secondary infections, we did not determine the serotype of the index infection, which, if unevenly distributed, could have biased our estimates of association. There are no reports in the literature specifically evaluating the effect of dengue serotypes on antioxidant responses; however, on the one hand, serotypes interact differently with the host’s immune system, eliciting inflammatory responses of varying intensity, and on the other, depending on the infection type different serotypes might increase the risk of developing more severe clinical manifestations [31,32]. Finally, there was an outlier observation among the controls that tended to underestimate the strength of the associations; however, it was deemed biologically plausible and, therefore, included in all the statistical models.

## 5. Conclusions

In conclusion, our work advanced the understanding of the role of the antioxidant response in patients with dengue through a methodologically robust study design. Specifically, our findings suggest that measuring TAS and SOD concentrations within the first days of DENV infection could contribute to identifying patients at increased risk of developing hypotensive and hemorrhagic complications. The early identification of at-risk individuals using these biomarkers could enable clinicians to provide closer monitoring of these patients and timely interventions to mitigate adverse outcomes. Further research is needed to confirm the potential prognostic role and clinical usefulness of the determination of TAS and SOD.

## Figures and Tables

**Table 1:** Baseline characteristics of dengue patients, by case-control status.

Characteristic	Controls (n = 88)	Cases (n = 44)	*p*
Age (years)	24.5 [14.0–37.0]	15.0 [10.0–21.5]	0.007
Age group			0.078
5–14 years	16 (27.3)	15 (43.2)	
≥15 years	64 (72.7)	25 (56.8)	
Male	46 (52.3)	21 (47.7)	0.713
Disease onset			0.389
48 h	18 (20.5)	5 (11.4)	
72 h	31 (35.2)	19 (43.2)	
94 h	39 (44.3)	20 (45.4)	
Primary infection	44 (52.4)	21 (47.7)	0.851
Warning signs *			
Persisting vomiting	0 (0.0)	3 (6.8)	0.035
Abdominal pain	32 (36.4)	24 (54.5)	0.561
Clinical fluid accumulation	22 (25.0)	6 (13.6)	0.181
Mucosal bleeding	0 (0.0)	9 (20.5)	<0.001
Lethargy or restlessness	45 (51.1)	29 (65.9)	0.137
Hepatomegaly	6 (6.8)	1 (2.3)	0.423
Laboratory			
Hemogram			
Hematocrit (%)	41.0 (3.5)	40.8 (3.7)	0.354
Leukocytes (per/mL)	3290 [2600–3800]	3290 [2600–3800]	0.940
Platelets (per/mL)	146,255 (44,709)	139,180 (46,713)	0.534
CRP (mg/dL)	9.6 [1.4–20.8]	11.9 [10.6–16.4]	0.407

* Persisting vomiting: ≥4 episodes during the last 24 h; clinical fluid accumulation: facial edema, signs of pleural or pericardial effusion, ascites, hydrocele, or lower limbs edema; mucosal bleeding: epistaxis or gingival hemorrhage; hepatomegaly: liver enlargement (>2 cm). Figures in each cell are absolute (relative) frequencies and means (SD) or medians [percentiles 25th–75th].

**Table 2:** Oxidative stress biomarkers measured in serum during the acute phase of dengue, by case-control status.

Characteristic	Controls (n = 88)	Cases (n = 44)	*p*
TAS (mM) *	1.7 [1.2–2.3]	2.1 [1.8–2.6]	0.057
SOD (U/mL) *	6.0 [5.0–7.1]	6.7 [5.6–7.8]	0.091
GPx (mmol/min/mL)	133.7 (24.1)	128.1 (20.2)	0.190

* Cases/controls: n = 27/32. Figures in each cell are means (SD) or medians [percentiles 25th–75th].

**Table 3:** Association between oxidative stress biomarkers and dengue complications *.

Biomarker	Piecewise Logistic Regression OR (95%CI)	Logistic Regression OR (95%CI)
TAS (mM) †		1.80 (0.89–3.64)
TAS < 3.5	2.46 (1.10–5.53)	
TAS ≥ 3.5	0.01 (0.00–406.3)	
Age	0.99 (0.96–1.02)	0.98 (0.95–1.02)
HL/AIC ‡	0.351/82.2	0.349/83.2
SOD (U/mL) †		1.04 (0.84–1.30)
SOD < 8.0	1.69 (1.01–2.83)	
SOD ≥ 8.0	0.77 (0.50–1.18)	
Age	0.99 (0.96–1.02)	0.98 (0.95–1.02)
HL/AIC	0.335/83.5	0.360/85.9
GPx (mmol/min/mL)		0.99 (0.98–1.01)
GPx < 103.0	1.06 (0.94–1.19)	
GPx ≥ 103.0	0.99 (0.97–1.01)	
Age	0.97 (0.95–1.00)	0.97 (0.95–1.00)
HL/AIC	0.343/167.8	0.357/167.1

* Hypotension or severe bleeding. † Cases/controls: n = 27/32. ‡ HL: *p*-value corresponding to the Hosmer-Lemeshow goodness of fit test; AIC: Akaike information criterion.

## Data Availability

The data supporting the findings of this study are available upon reasonable request. Due to privacy and ethical restrictions, the data cannot be made publicly available.

## Supplementary Materials

The following supporting information can be downloaded at: https://www.mdpi.com/article/10.3390/tropicalmed10010014/s1, Supplementary Table S1. Association between oxidative stress biomarkers and dengue complications * among confirmed dengue cases.
