# Supplementary material for: Antioxidant Response as a Candidate Prognostic Factor for Dengue Hypotensive and Hemorrhagic Complications: Results from a Nested Case-Control Study in Colombia
Source: Trop Med Infect Dis. 2025 Jan 4;10(1):14. doi: 10.3390/tropicalmed10010014 (PMC11769034; doi:10.3390/tropicalmed10010014)
Supplement: Supplementary file 1 [file tropicalmed-10-00014-s001.zip › tropicalmed-3373862-supplementary.pdf]

Supplementary Table S1. Association between oxidative stress biomarkers and dengue complications* among confirmed dengue cases.

Biomarker	Piecewise Logistic regression OR (95%CI)	Logistic regression OR (95%CI)
**TAS (mM)†**		1.68 (0.83 - 3.39)
TAS<3.5	2.27 (1.01 - 5.07)	…
TAS≥3.5	0.01 (0.00 - 375.2)	…
Age	0.99 (0.95 - 1.02)	0.98 (0.95 - 1.02)
HL / AIC¶	0.346 / 77.8	0.348 / 78.6
**SOD (U/mL)†**		1.01 (0.81 - 1.27)
SOD<8.0	1.53 (0.89 - 2.61)	…
SOD≥8.0	0.79 (0.52 - 1.20)	…
Age	0.99 (0.96 - 1.02)	0.98 (0.95 - 1.02)
HL / AIC¶	0.331 / 79.9	0.354 / 80.8
**GPx (mmol/min/ml)‡**		1.00 (0.98 - 1.02)
GPx<103.0	1.11 (0.96 - 1.28)	…
GPx≥103.0	0.99 (0.97 - 1.01)	…
Age	0.98 (0.95 - 1.00)	0.97 (0.95 - 1.00)
HL / AIC¶	0.363 / 155.4	0.370 / 155.8

* Hypotension or severe bleeding. † Cases/controls: n=26/29. ‡ Cases/controls: n=42/77. ¶ HL: P-value corresponding to the Hosmer-Lemeshow goodness of fit test; AIC: Akaike information criterion.
